# Off-Label Ustekinumab and Vedolizumab in Pediatric Anti-TNFα Refractory IBD: Therapeutic Drug Monitoring Insights from a Case Series

**DOI:** 10.3390/ph19010154

**Published:** 2026-01-15

**Authors:** Stefania Cheli, Giulia Mosini, Vera Battini, Carla Carnovale, Sonia Radice, Marta Lebiu, Alessandro Cattoni, Giovanna Zuin, Emilio Clementi

**Affiliations:** 1 ICPS, Pharmacovigilance & Clinical Research, Department of Biomedical and Clinical Sciences, University Hospital Luigi Sacco, ASST Fatebenefratelli Sacco, Università degli Studi di Milano, 20157 Milan, Italy; giulia.mosini@asst-fbf-sacco.it (G.M.); vera.battini@unimi.it (V.B.); carla.carnovale@unimi.it (C.C.); sonia.radice@unimi.it (S.R.); emilio.clementi@unimi.it (E.C.); 2 Pediatric Department Fondazione IRCCS San Gerardo dei Tintori, 20900 Monza, Italy; marta.lebiu@irccs-sangerardo.it (M.L.); alessandro.cattoni@irccs-sangerardo.it (A.C.); giovanna.zuin@irccs-sangerardo.it (G.Z.); 3 Scientific Institute, IRCCS E. Medea, 23842 Bosisio Parini, Italy

**Keywords:** pediatric inflammatory bowel disease, vedolizumab, ustekinumab, therapeutic drug monitoring, case series

## Abstract

**Background**: Vedolizumab and ustekinumab are increasingly used off-label in pediatric inflammatory bowel disease (IBD) unresponsive or refractory to anti–TNFα therapy. Despite their increasing use in clinical practice, evidence in the pediatric population remains limited, especially regarding therapeutic exposure thresholds and the clinical utility of therapeutic drug monitoring (TDM). **Methods**: We report a series of five pediatric cases with Crohn’s disease or ulcerative colitis treated with ustekinumab or vedolizumab after anti-TNFα failure. Trough drug concentrations, anti-drug antibodies (ADAs), clinical scores (PCDAI/PUCAI), biomarkers (fecal calprotectin, C-reactive protein), and endoscopic findings were assessed longitudinally. **Results**: In all cases, we observed recurrent discordance between clinical indices (PCDAI/PUCAI), biochemical markers, and endoscopic activity. Clinical improvement frequently correlated with trough concentrations above commonly cited adult-derived reference ranges (>15 µg/mL for vedolizumab; >3 µg/mL for ustekinumab), although this alignment was not uniform across patients. Notably, one patient developed high-titre ADAs with undetectable ustekinumab levels, yet remained clinically stable, suggesting substantial interindividual variability in pharmacokinetics, immunogenicity, and disease control. **Conclusions**: Ustekinumab and vedolizumab are promising off-label options for pediatric refractory IBD. In this case series, TDM contributed to the interpretation of pharmacokinetic variability and immunogenicity, offering contextual insights that may support dose adjustments and therapeutic decision-making. Integrating TDM with clinical, biochemical, and endoscopic monitoring may improve optimize individualized treatment in this complex and vulnerable patient group.

## 1. Introduction

The introduction of anti–tumor necrosis factor α therapy (anti-TNFα) has fundamentally transformed the management of inflammatory bowel disease (IBD), by achieving satisfactory clinical responses in a significant portion of patients, including long-term remission [1,2,3]. Over the past decades, the cumulative five-year exposure to anti-TNFα agents has risen to more than 50%, reflecting their increasingly central role in clinical practice. This widespread use has been linked to a reduction in the risk of intestinal resections, with cohort studies reporting significantly lower hazard ratios for surgery among patients receiving early anti-TNFα therapy [4,5]. Moreover, in patients with symptomatic strictures, anti-TNFα treatment may delay or, in some cases, avert the need for operative intervention [6]. Nonetheless, the benefit in terms of preventing stricture formation is not consistently demonstrated across studies, and the overall decline in resection rates observed in recent decades is likely influenced by additional factors, including earlier diagnosis, multidisciplinary management, and optimized therapeutic strategies [7,8]. Despite these advances, important therapeutic challenges persist, particularly in pediatric patients who are refractory or intolerant to anti-TNFα therapy, underscoring the ongoing need for alternative therapeutic options. The most considered alternatives for these vulnerable patients are vedolizumab, a monoclonal antibody that blocks T-cell trafficking to the gastrointestinal tract, and ustekinumab, an inhibitor of interleukin-12 and interleukin-23 (IL-12/23) [9]. Emerging evidence suggests that these agents may be effective in children with severe treatment-refractory disease, although the available data remain limited [10,11]. Therapeutic drug monitoring (TDM) has been proposed as a strategy to optimize biologic therapy in this complex population [12,13]. By measuring drug concentrations and detecting ADAs, TDM allow for individualized dose adjustments and the early identification of immunogenicity. However, for newer biologics such as ustekinumab and vedolizumab, the available literature provides limited evidence regarding optimal dosing strategies, target drug concentrations, and the achievement of adequate trough levels [12,14,15,16]. In adult patients, achieving therapeutic drug levels of these agents has been associated with higher rates of clinical remission and sustained response, suggesting potential benefits might be achievable in pediatric populations [17,18,19,20]. Consequently, the application of TDM in children could therefore facilitate timely identification of primary non-response or secondary loss of response, allowing dose optimization or early therapeutic switching.

In this context, we present our experience with five pediatric patients with IBD who were refractory to anti-TNFα therapy and subsequently received vedolizumab or ustekinumab. This case series provided additional insights into the limited existing evidence regarding the off-label use of these therapies and highlights the potential role of TDM in guiding personalized treatment strategies for complex and vulnerable pediatric cases [21].

## 2. Results 

A total of five patients, four of which were male, were initiated on vedolizumab or ustekinumab at a median age of 15 years (range 11.5–16). Three patients had UC and 2 had CD, all five had undergone therapy with anti-TNFα, immunosuppressant as well as mesalazine and corticosteroids for UC before starting vedolizumab or ustekinumab. Three of the patients underwent endoscopy during follow-up and no side effects of vedolizumab or ustekinumab were observed. Baseline clinical characteristics are summarized in Table 1.

### 2.1. Case 1

**Figure 1:**
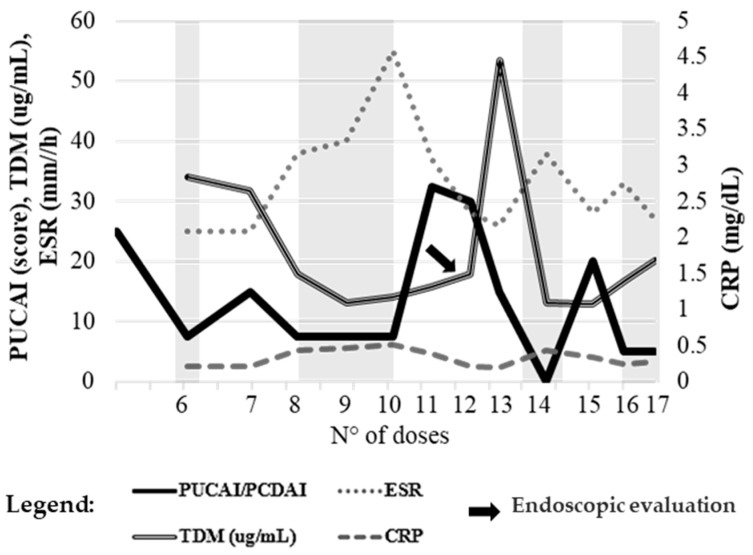
Case 1. Temporal trends of PUCAI score, plasma trough concentrations (TDM), and inflammatory markers (ESR, CRP) during vedolizumab therapy. The arrow indicates the time point of endoscopic evaluation. Reference values: ESR ≤ 10 mm/h, CRP ≤ 0.5 mg/dL, TDM ≥ 15 µg/mL, PUCAI ≤ 10. The left *y*-axis reports PUCAI, ESR, and TDM values; the right *y*-axis shows CRP levels. The x-axis represents the number of drug administrations, used to accurately reflect patient follow-up despite non-uniform time intervals resulting from individualized dosing schedules and the timing of biochemical and pharmacokinetic assessments. Gray-shaded areas indicate intervals during which the PUCAI score was <10, corresponding to periods of clinical remission.

A 10-year-old boy with UC initiated vedolizumab (5.5 mg/kg) with a baseline PUCAI of 25. As shown in Figure 1, clinical remission was achieved between dose 6 and 10, although a marked rise in fecal calprotectin (4925 µg/g) and ESR (55 mm/h) emerged around dose 9. During the same period, vedolizumab trough levels declined (13.1–14.1 µg/mL), indicating suboptimal exposure.

A clinical relapse occurred after dose 11 and 12, prompting interval shortening to every five weeks. Despite this adjustment, PUCAI, inflammatory markers, and trough levels remained discordant. An endoscopy performed shortly after dose 12 (as indicated by the arrow in Figure 1), confirmed active mucosal inflammation, with a Mayo endoscopic subscore of 2 in the colon and 3 in the sigmoid and rectum. These findings suggested persistent underexposure and ongoing disease activity despite dose optimization.

Vedolizumab was briefly intensified to a 4-weekly regimen, but given the lack of sustained improvement, therapy was discontinued and ustekinumab initiated, resulting in both clinical and endoscopic remission.

### 2.2. Case 2

**Figure 2:**
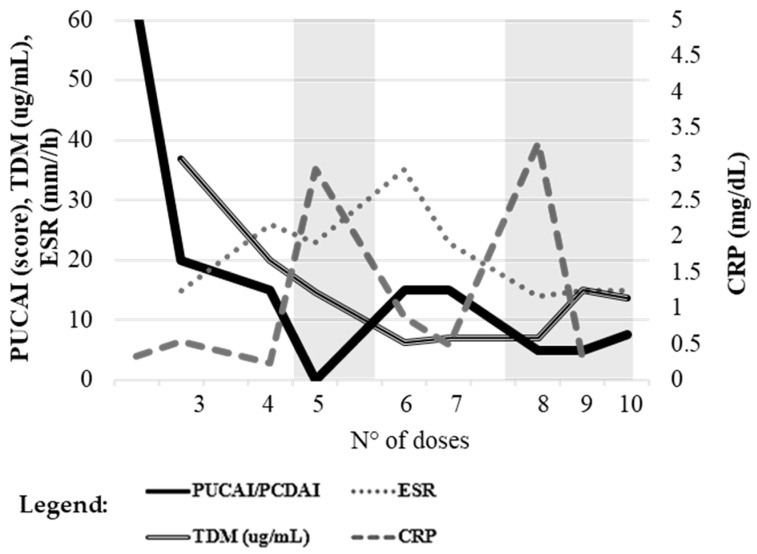
Case 2. Temporal trend of PUCAI score, plasma trough concentrations, and inflammatory markers (ESR, CRP) across vedolizumab treatment. No endoscopic evaluation was performed. Reference values: ESR ≤ 10 mm/h, CRP ≤ 0.5 mg/dL, TDM ≥ 15 µg/mL, PUCAI ≤ 10. The left *y*-axis reports PUCAI, ESR, and TDM values; the right *y*-axis shows CRP levels. The x-axis represents the number of drug administrations, used to accurately reflect patient follow-up despite non-uniform time intervals resulting from individualized dosing schedules and the timing of biochemical and pharmacokinetic assessments. Gray-shaded areas indicate intervals during which the PUCAI score was <10, corresponding to periods of clinical remission.

A 17-year-old patient with severe UC (PUCAI 65) initiated vedolizumab. As shown in Figure 2, the PUCAI score rapidly decreased, reaching 20 during induction and clinical remission (PUCAI < 10) from the fifth dose onward. Despite sustained symptomatic improvement, inflammatory markers remained consistently elevated throughout follow-up, with fecal calprotectin (764 µg/g), ESR (15 mm/h) and CRP (0.53 mg/dL) fluctuating but never normalizing. Albumin and hemoglobin remained stable.

Vedolizumab trough levels were initially subtherapeutic in early maintenance (from dose 6), remaining below 10 µg/mL, but progressively increased over time, reaching 13.6 µg/mL at the tenth dose, coinciding with stable clinical remission (PUCAI 7.5). No endoscopic assessment was performed, but the persistent elevation of inflammatory biomarkers despite clinical remission suggests a discordance between symptomatic control and mucosal healing.

Near transition to adult care at age 18, mesalazine was introduced, and vedolizumab dosing was temporarily intensified in response to mild rectal bleeding occurring six weeks after a standard dosing interval.

### 2.3. Case 3

**Figure 3:**
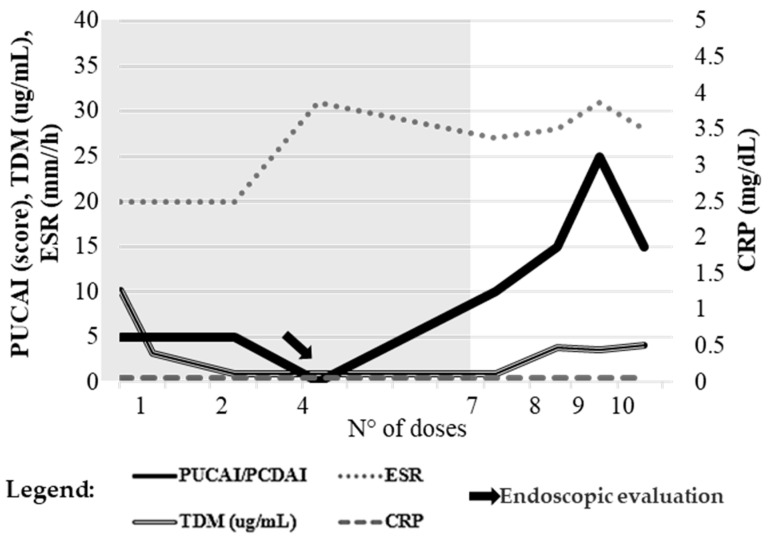
Case 3. Temporal trends of PUCAI score, plasma trough concentrations (TDM), and inflammatory markers (ESR, CRP) during ustekinumab therapy. The arrow marks the time point of endoscopic evaluation. Reference values: ESR ≤ 10 mm/h, CRP ≤ 0.5 mg/dL, TDM ≥ 3 µg/mL, PUCAI ≤ 10. The left *y*-axis reports PUCAI, ESR, and TDM values; the right *y*-axis shows CRP levels. The x-axis represents the number of drug administrations, used to accurately reflect patient follow-up despite non-uniform time intervals resulting from individualized dosing schedules and the timing of biochemical and pharmacokinetic assessments. Gray-shaded areas indicate intervals during which the PUCAI score was <10, corresponding to periods of clinical remission.

A 13-year-old patient with chronic active UC, autoimmune hepatitis, and primary sclerosing cholangitis initiated ustekinumab as rescue therapy prior to a potential colectomy. As shown in Figure 3, despite a low baseline PUCAI of 5, the clinical course was unstable, with fecal calprotectin progressively increasing from induction through the ninth dose (from 177 to 697 µg/g). The PUCAI rose to 25 at dose 9, indicating a flare, before partially improving at dose 10.

Ustekinumab trough levels, initially within the therapeutic range, declined to subtherapeutic values between the second and seventh doses (TDM < 3 µg/mL). An endoscopy performed shortly after dose 4 confirmed active mucosal inflammation, with diffuse loss of vascular pattern, mucosal atrophy, and spontaneous bleeding in the right colon. Following dose intensification to a 4-weekly regimen, trough levels increased, but inflammatory markers, particularly fecal calprotectin and ESR, continued to rise from dose 7 onward (from 493 to 697 µg/g; from 27 to 31 mm/h, respectively).

Other laboratory parameters, including CRP, hemoglobin, and albumin, remained stable. Overall, the findings indicated persistent mucosal inflammation, biochemical activity, and variable drug exposure despite therapeutic optimization. The patient ultimately underwent colectomy.

### 2.4. Case 4

**Figure 4:**
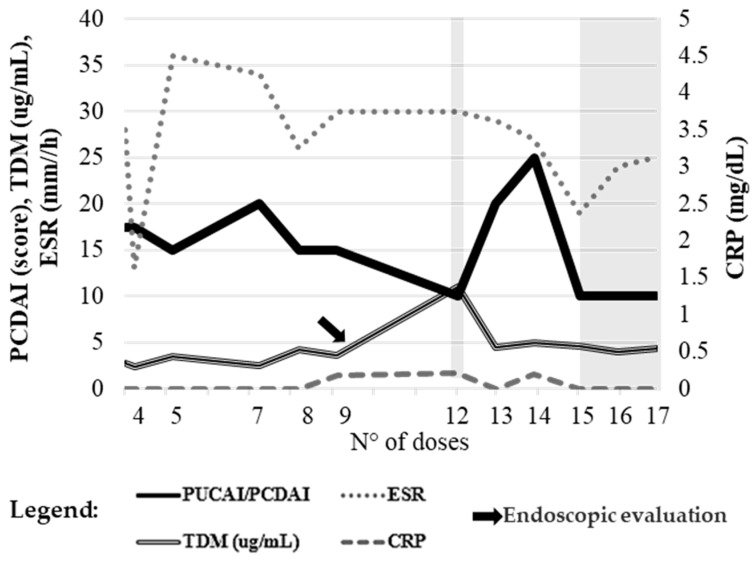
Case 4. Temporal trends of PCDAI score, plasma trough concentrations, and inflammatory markers (ESR, CRP) during ustekinumab therapy. The arrow indicates endoscopic evaluation. Reference values: ESR ≤ 10 mm/h, CRP ≤ 0.5 mg/dL, TDM ≥ 3 µg/mL, PCDAI ≤ 10. The left *y*-axis reports PUCAI, ESR, and TDM values; the right *y*-axis shows CRP levels. The x-axis represents the number of drug administrations, used to accurately reflect patient follow-up despite non-uniform time intervals resulting from individualized dosing schedules and the timing of biochemical and pharmacokinetic assessments. Gray-shaded areas indicate intervals during which the PUCAI score was <10, corresponding to periods of clinical remission.

A 15-year-old patient with ileocolonic and perianal CD initiated ustekinumab with a baseline PCDAI of 37.5. As shown in Figure 4, clinical improvement was observed during maintenance, with the PCDAI decreasing to 10 by dose 12. Intermittent increases to 20–25 at doses 13 and 14 indicated episodic disease exacerbations before stabilizing again at 10.

Inflammatory markers showed variable activity: ESR fluctuated with peaks in early maintenance (from 13 to 36 mm/h), while fecal calprotectin remained stable initially but rose markedly at dose 17 (1800 µg/g), suggesting a flare or suboptimal response (Figure 4). Hemoglobin, albumin, and CRP were largely stable, although a drop in hemoglobin at dose 17 (8.9 g/dL) was consistent with active disease.

Endoscopy performed after dose 9 revealed persistent inflammation in the left colon, sigmoid, and rectum, with erythema, loss of vascular pattern, and small fibrin-covered ulcerations, indicating incomplete mucosal healing. Due to ongoing disease activity, adalimumab was initiated with weekly maintenance dosing. A subsequent endoscopy again demonstrated persistent inflammation.

The patient was therefore switched to risankizumab, which led to progressive clinical and biochemical improvement, including a marked reduction in fecal calprotectin.

### 2.5. Case 5

**Figure 5:**
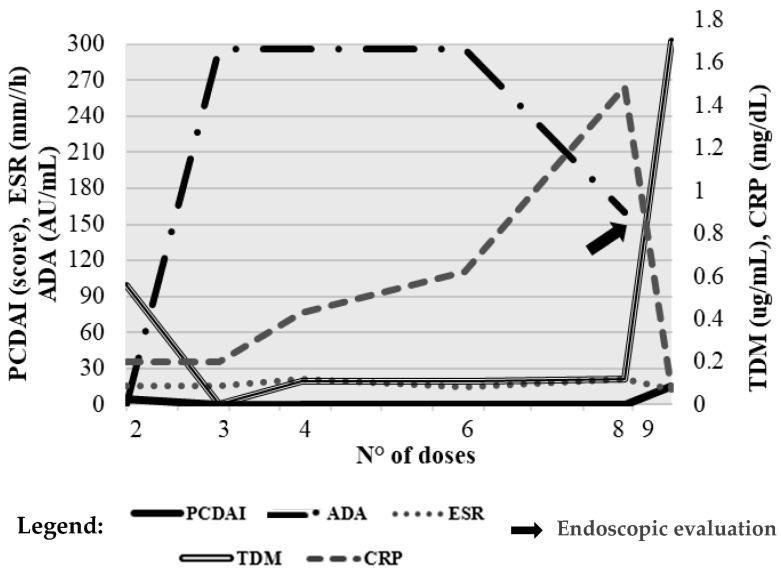
Case 5. Temporal trends of plasma trough concentrations (TDM), clinical disease activity (PCDAI), inflammatory markers (ESR, CRP), and immunogenicity (ADA) during ustekinumab therapy. Reference values: ESR ≤ 10 mm/h, CRP ≤ 0.5 mg/dL, TDM ≥ 3 µg/mL, PCDAI ≤ 10 and ADA ≥ 3.5 AU/mL. The left *y*-axis reports PCDAI, ESR, and ADA values; the right *y*-axis shows TDM and CRP levels. The x-axis represents the number of drug administrations, used to accurately reflect patient follow-up despite non-uniform time intervals resulting from individualized dosing schedules and the timing of biochemical and pharmacokinetic assessments. Gray-shaded areas indicate intervals during which the PUCAI score was <10, corresponding to periods of clinical remission.

A 15-year-old patient with CD initiated ustekinumab after failure of multiple anti-TNFα agents, which had been discontinued due to severe psoriasis. At baseline, the patient was in clinical remission (PCDAI 0). As shown in Figure 5, a marked discrepancy emerged throughout follow-up between ustekinumab trough levels and clinical status: despite persistently low or undetectable concentrations, associated with high-titre anti-drug antibodies (>296 AU/mL), the patient maintained low disease activity, with PCDAI values ranging from 0 to 15.

Inflammatory markers showed only mild variability, and hemoglobin and albumin remained stable. However, fecal calprotectin rose markedly at dose 9 (1150 µg/g), indicating active intestinal inflammation despite minimal symptoms (PCDAI 15). This discordance highlighted the impact of immunogenicity and the need for integrated interpretation of clinical, biochemical, and pharmacokinetic data.

Approximately 10 months into therapy, the patient developed weight loss, and endoscopy revealed active mucosal inflammation, suggesting subclinical disease progression. Given the presence of high-titre antibodies, subtherapeutic drug levels, and confirmed endoscopic activity, ustekinumab was discontinued and risankizumab initiated, resulting in progressive clinical and biochemical improvement.

### 2.6. Integrated Overview of Clinical Trajectories and TDM-Informed Decisions

To facilitate comparison across cases and provide a comprehensive overview of the clinical trajectories, Table 2 summarizes the key elements of each patient’s therapeutic course, including the index biologic, median trough concentrations with corresponding ranges, immunogenicity status, TDM-informed therapeutic modifications, and final outcomes. This synthesis illustrates how trough levels and ADAs results were interpreted alongside clinical, biochemical, and, when available, endoscopic assessments. The table also reflects the heterogeneity of pharmacokinetic profiles, dosing adjustments, and response patterns observed in this cohort.

## 3. Discussion

This case series provides a descriptive overview of the real-world use of vedolizumab and ustekinumab in pediatric patients with IBD refractory to anti-TNFα therapy. Although limited by sample size, the cases illustrate the complexity of managing second- and third-line biologics in children, particularly regarding pharmacokinetic variability, immunogenicity, and the frequent discordance between clinical symptoms, biochemical markers, and mucosal disease activity. A recurring observation across the presented cases was the discordance between clinical symptoms and biochemical or endoscopic findings. Several patients exhibited low PCDAI or PUCAI scores despite elevated fecal calprotectin or active endoscopic inflammation. This highlights the limitations of symptom-based assessment alone and supports the importance of a multidimensional monitoring approach that combines clinical indices with biomarkers and, when feasible, endoscopy evaluation.

Within this complex landscape, TDM contributed to the interpretation of treatment response, particularly when subtherapeutic exposure or suspected immunogenicity was present. In Cases 1–3, TDM supported dose escalation strategies in response to subtherapeutic trough concentration. These observations align with emerging pediatric data suggesting that standard dosing regimens may be insufficient for a substantial proportion of children, who often require shortened intervals or higher maintenance doses to achieve adequate exposure. However, given that current exposure thresholds are largely extrapolated from adult cohorts, the interpretation of TDM in children must remain cautious and contextual. In contrast, Case 5 demonstrated high-titre anti-ustekinumab antibodies associated with undetectable drug levels, despite the patient maintaining a stable clinical condition. This case may illustrate the complex interplay between pharmacokinetics, immunogenicity, and disease control. While ADAs are generally associated with reduced therapeutic efficacy, their presence does not necessarily predict clinical failure, suggesting that alternative mechanisms may sustain remission in certain patients. Several mechanisms may contribute to this variability, including differences in antibody neutralizing capacity, residual tissue-level drug activity, or disease phenotypes less dependent on IL-12/23 signaling. Such findings emphasize that TDM and ADA testing, while informative, cannot be interpreted in isolation and must be integrated with clinical, biochemical, and endoscopic data.

Endoscopic findings in several cases (e.g., Cases 1 and 4) confirmed persistent mucosal inflammation despite apparent clinical improvement and/or increased drug levels, reinforcing the notion that clinical remission alone does not necessarily equate to disease control [22,23,24,25]. These observations may align with previous reports suggesting that some patients in apparent remission could still to exhibit subclinical disease activity detectable only through biomarkers or endoscopy [26,27,28]. Given that endoscopy is invasive and not routinely performable during follow-up, the identification of reliable non-invasive markers of mucosal healing remains a clinical priority [22,23,26,28]. Fecal calprotectin remains the most widely used surrogate, but its limitations in pediatrics are well recognized [27,29]. In this regard, TDM could provide complementary information, particularly when integrated with ADA testing and clinical scores.

Therapeutic thresholds for ustekinumab and vedolizumab in the pediatric population remain to be clearly established [30,31,32,33]. Current target concentrations are largely extrapolated from adult data, but pediatric pharmacokinetics and pharmacodynamics may differ due to developmental, immunological, and disease-related factors [33,34,35,36]. Data from recent pediatric cohorts indicate that 35–50% of patients may exhibit subtherapeutic trough concentrations during early treatment, highlighting the potential value of proactive TDM to guide early dose optimization [32,37]. Several studies have also shown that standard dosing regimens are frequently inadequate in children, necessitating intravenous induction, dose intensification, or shortened dosing intervals to achieve therapeutic targets [10,32,37]. These observations are consistent with our clinical experience, in which proactive adjustments were often required. Notably, Case 5 maintained clinical stability despite undetectable drug levels, suggesting considerable inter-individual variability in pharmacodynamic response and underscoring the need for personalized treatment approaches.

The clinical relevance of ADAs, though infrequent with ustekinumab, requires particular attention. In Case 5, persistent high-titre ADAs were observed alongside sustained clinical remission, suggesting that the relationship between immunogenicity and treatment response is not linear. While persistent ADAs have generally been associated with lower drug concentrations and poorer clinical outcomes, transient ADAs appear to exert only a limited and often clinically irrelevant impact [20,38]. This discrepancy could reflect compensatory immunological mechanisms, residual drug activity at tissue level, or a disease phenotype less dependent on IL-12/23 pathways. It also underscores the importance of interpreting ADA results within an integrated clinical and biochemical context, rather than relying solely on pharmacokinetic data.

The interpretation of TDM in this setting is also subject to several important limitations. Assay-related variability must be considered, as ELISA-based platforms differ in analytical sensitivity, calibration curves, and drug–antibody interference, potentially affecting the comparability of trough concentrations across studies. Inflammation-driven changes in drug clearance, particularly during active disease, may lead to lower trough levels independently of dosing adequacy, complicating the distinction between true underexposure and disease-related pharmacokinetic alterations. Moreover, pediatric pharmacokinetics are strongly influenced by inflammatory burden, nutritional status, and concomitant therapies, all of which may alter drug clearance and confound the interpretation of trough concentrations. The absence of validated pediatric therapeutic thresholds further limits the ability to define exposure–response relationships with confidence. These factors highlight the need for cautious, context-dependent interpretation of TDM results.

Overall, our findings suggest that the combined use of TDM and biomarker assessment may support clinical decision-making in selected pediatric patients treated with vedolizumab or ustekinumab, particularly when the clinical picture is atypical or the therapeutic response appears suboptimal. Although the potential value of TDM is increasingly recognized, its application is currently more feasible during induction, when pharmacokinetic variability is greatest and therapeutic objectives are clearly defined. In contrast, monitoring during maintenance often remains reactive, constrained by the absence of reliable surrogate markers of disease activity and by the practical limitations of routine pediatric care.

Interpretation of the TDM results in this setting requires careful consideration of multiple confounding factors that influence both drug exposure and clinical response. Disease phenotype (Crohn’s disease vs. ulcerative colitis), concomitant therapies such as corticosteroids or immunomodulators, and variability in dosing regimens, including induction strategies and interval adjustments, may all contribute to heterogeneity in drug exposure and therapeutic response. These factors can obscure direct associations between trough concentrations, immunogenicity, and clinical status, underscoring the need for prospective studies designed to account for these variables when evaluating the utility of TDM in pediatric IBD.

The main limitations of this case series include its small sample size and retrospective design, which limit generalizability. Nevertheless, the cases presented reflect real-world complexities in the management of refractory pediatric IBD and raise the hypothesis that integrating TDM and immunogenicity assessment into clinical evaluation may help refine individualized therapeutic strategies.

## 4. Materials and Methods

We discuss five clinical cases of pediatric patients with anti-TNFα-refractory IBD, who received off-label treatment with ustekinumab or vedolizumab. Patients were followed from May 2022 to June 2023 with monthly or bimonthly follow-up visits during which clinical and biochemical data were collected. Patients had a prior diagnosis of Crohn’s disease (CD) or ulcerative colitis (UC) based on the revised Porto criteria of the European Society for Pediatric Gastroenterology, Hepatology, and Nutrition (ESPGHAN) [39]. Demographic and clinical data were collected, including age at diagnosis, disease localization, and behavior according to the Paris classification [40]. For each patient, clinical disease activity was assessed using the Pediatric Crohn’s Disease Activity Index (PCDAI) for CD and the Pediatric Ulcerative Colitis Activity Index (PUCAI) for UC at baseline (prior to ustekinumab or vedolizumab therapy initiation), at each TDM assessment, and at the end of follow-up. Clinical remission was defined as PCDAI and PUCAI scores ≤ 10. Clinical response was defined as a decrease in PCDAI by 12.5 points or in PUCAI by 10 points. Laboratory data, including serum hemoglobin, C-reactive protein (CRP), erythrocyte sedimentation rate (ESR), albumin, and fecal calprotectin (FC), were collected for all patients. For the descriptive clinical interpretation of TDM results, trough concentrations (TC) > 15 µg/mL for vedolizumab and >3 µg/mL for ustekinumab were considered indicative of adequate drug exposure. These thresholds were selected based on established exposure–response data in adult cohorts and emerging evidence in the pediatric population [10,20,37], serving as clinical reference points to guide dose optimization rather than absolute therapeutic targets, as definitive pediatric thresholds are yet to be fully established.

Data on clinical management (i.e., treatment intensification, agents like steroids, non-steroid anti-inflammatory drugs) as well as available endoscopic assessments were recorded.

### 4.1. Ethics Statement

This study was conducted using data previously collected for clinical purposes and subsequently anonymized, in full compliance with the EU General Data Protection Regulation (GDPR, Regulation 2016/679). All patients were enrolled in the IBD Registry of the Italian Society of Pediatric Gastroenterology, Hepatology and Nutrition (SIGENP). The study was carried out in accordance with the principles of the Declaration of Helsinki.

### 4.2. Assessment of Plasma Trough Concentrations and Anti-Drug Antibodies

Plasma trough concentrations of vedolizumab and ustekinumab, as well as their respective ADAs, were quantified using enzyme-linked immunosorbent assays (ELISAs). Plasma concentrations of vedolizumab and ustekinumab were obtained immediately before drug administration at steady state. Plasma samples were collected at each visit and analyzed retrospectively following the final follow-up assessment. Concentrations were quantified using CHORUS Promonitor kits on the CHORUS TRIO instrument (Diesse Diagnostica Senese, Siena, Italy), according to the manufacturer’s instructions. The CHORUS TRIO instrument reports results in µg/mL, calculated based on a batch-dependent curve stored within the system. The methodological assessment of plasma vedolizumab and ustekinumab concentrations results is provided Appendix A, Table A1. CHORUS Promonitor anti-vedolizumab and anti-ustekinumab kits were used to detect ADAs against the respective drugs. The interpretation of ADA test results is shown in Appendix B, Table A2.

### 4.3. Statistical Analysis

A descriptive statistical approach was employed to evaluate the clinical and biochemical parameters of this case series. Data were retrospectively collected from electronic medical records to explore longitudinal trends and potential discrepancies between clinical indices and biochemical markers.

Given the small sample size (n = 5) and the high degree of clinical heterogeneity among patients, specifically regarding disease phenotype, treatment history, and dosing schedules; inferential statistical analyses (e.g., *p*-values) were not performed, as they would lack sufficient power and could lead to misleading interpretations.

Descriptive statistics, including medians and ranges (IQR), were utilized to summarize the data where appropriate. Temporal trends and correlations between variables, including clinical indices (PUCAI/PCDAI), biochemical markers (CRP, FC, ESR), and drug trough concentrations (TC), were assessed through visual inspection of plotted data and qualitative comparisons. This case-by-case longitudinal evaluation was prioritized to provide a detailed and transparent representation of each patient’s clinical course and treatment response.

## 5. Conclusions

This case series provides exploratory observations on the off-label use of ustekinumab and vedolizumab in pediatric patients with IBD refractory to anti-TNFα agents. Although limited by sample size and retrospective design, the cases illustrate the clinical heterogeneity of this population and the challenges inherent in managing second- and third-line biologic treatments.

Within this context, TDM contributed to the interpretation of treatment response, particularly during induction and in cases of suspected underexposure or immunogenicity. Integrating TDM with clinical indices, biomarkers, and, where feasible, endoscopic evaluation, TDM may support more informed and individualized therapeutic decisions. At the same time, the observation of sustained clinical stability despite undetectable drug levels in one patient illustrates the need for caution when interpreting pharmacokinetic data in isolation and highlights the importance of a multidimensional approach.

Current therapeutic thresholds for ustekinumab and vedolizumab in pediatric populations remain largely extrapolated from adult data. Our experience suggests that proactive dose optimization and personalized regimens could be areas for future investigation. Prospective studies are essential to establish pediatric specific exposure targets, validate early pharmacodynamic markers, and clarify the clinical relevance of anti-drug antibodies.

In summary, TDM represents a promising practical tool to support clinical decision-making in pediatric refractory IBD, particularly for complex cases requiring tailored interventions. Future research should focus on defining treat-to-target strategies that integrate pharmacokinetics, immunogenicity, and mucosal healing to advance precision medicine in this vulnerable population.

## Figures and Tables

**Table 1:** Baseline clinical characteristics of patients.

ID	Sex	BMI	IBD Type	Paris Classification	PCDAI/ PUCAI (Therapy Onset)	Therapy	Dose (mg/kg)	Concomitant Therapy
Case 1	M	19.2	UC	A1aE4S1G0	25	VDZ	5.5	Mesalazine
Case 2	M	16.7	UC	A1bE4S0G0	60–65	VDZ	5.5	Corticosteroids ^(1)^, azathioprine ^(1,2)^, mesalazine ^(3)^
Case 3	M	18.9	UC	A1bE1S0G0	5	USK	6	Mercaptopurine, vancomycin
Case 4	F	21.5	CD	A1bL2B1pG0	37.5	USK	5	Mesalazine ^(1)^, adalimumab ^(2)^
Case 5	M	18.7	CD	A1bL4aB2G1	0	USK	1.5	-

^(1)^ First concomitant therapy; ^(2)^ Second concomitant therapy; ^(3)^ Third concomitant therapy. IBD: Inflammatory Bowel Disease; PCDAI: Pediatric Crohn’s Disease Activity Index; PUCAI: Pediatric Ulcerative Colitis Activity Index; VDZ: vedolizumab; USK: ustekinumab; CD: Crohn’s Disease; UC: Ulcerative Colitis.

**Table 2:** Summary of TDM Findings, treatment decisions, and clinical outcomes.

Case	Drug	Trough Level (µg/mL) Median [Range]	ADA (AU/mL)	Key Clinical Decision	Outcome
1	VDZ	17.35 [14–23]	<8	Dose intensification → switch to USK	Clinical + endoscopic remission
2	VDZ	7 [10–14]	<8	Temporary dosing intensification	Clinical remission, biomarkers elevated
3	USK	3.7 [3–4]	<8	Intensification → colectomy	Persistent disease activity → Surgery
4	USK	4.5 [4–5]	neg	Switch to ADM → switch to RZB	Progressive clinical and biochemical improvement
**5**	USK	undetectable	>296	Switch to RZB	Favorable clinical and biochemical responses

Abbreviations: ADA: anti-drug antibody; VDZ, vedolizumab; USK, ustekinumab; ADM: adalimumab; RZB: risankizumab.

## Data Availability

The original contributions presented in this study are included in the article. Further inquiries can be directed to the corresponding author.

## Abbreviations

The following abbreviations are used in this manuscript:

ADA	Anti-Drug Antibody
CD	Crohn’s Disease
CRP	C-Reactive Protein
ESPGHAN	European Society for Pediatric Gastroenterology, Hepatology and Nutrition
ESR	Erythrocyte Sedimentation Rate
FC	Fecal Calprotectin
GDPR	General Data Protection Regulation
IBD	Inflammatory Bowel Disease
IL	Interleukin
IQR	Interquartile Range
PCDAI	Pediatric Crohn’s Disease Activity Index
PUCAI	Pediatric Ulcerative Colitis Activity Index
TDM	Therapeutic Drug Monitoring
TNFα	Tumor Necrosis Factor Alpha
UC	Ulcerative Colitis

## Appendix A

This appendix provides the methodological criteria for evaluating vedolizumab (VDZ) and ustekinumab (USK) trough concentrations obtained using CHORUS Promonitor assays. Equivocal results correspond to values between the limit of detection (<1.1 µg/mL for VDZ, <0.10 µg/mL for USK) and the lower limit of quantitation (1.5 µg/mL for VDZ, 0.19 µg/mL for USK).

**Table A1:** Methodological evaluation of plasma drug concentration results.

Results	VDZ Concentration	USK Concentration
Positive	≥1.5 µg/mL	≥0.19 µg/mL
Negative	≤1.1 µg/mL	≤0.10 µg/mL
Equivocal	1.2–1.4 µg/mL	0.11–0.18 µg/mL

## Appendix B

This appendix provides the interpretation criteria for anti-vedolizumab (anti-VDZ) and anti-ustekinumab (anti-USK) antibody concentrations obtained using CHORUS Promonitor assays. Equivocal results correspond to values between the limit of detection (<8.0 AU/mL for anti-VDZ, <1.5 AU/mL for anti-USK) and the lower limit of quantitation (9.7 AU/mL for anti-VDZ, 3.5 AU/mL for anti-USK).

**Table A2:** ADA test results interpretation.

Results	VDZ Concentration	USK Concentration
Positive	≥9.7 AU/mL	≥3.5 AU/mL
Negative	≤8.0 AU/mL	≤1.5 AU/mL
Equivocal	8.1—9.6 AU/mL	1.6–3.4 AU/mL
